# Impediments to transforming the healthcare delivery system: shifting the paradigm from provider centric to patient centric

**DOI:** 10.3389/frhs.2026.1729616

**Published:** 2026-03-04

**Authors:** Elizabeth A. Regan, Manasa Devi Chinta

**Affiliations:** 1 Health Informatics Lab, Integrated Information Technology Department, Molinaroli College of Engineering and Computing, University of South Carolina, Columbia, SC, United States; 2 Health Informatics, Integrated Information Technology Department, Molinaroli College of Engineering and Computing, University of South Carolina, Columbia, SC, United States

**Keywords:** design science, digital transformation, healthcare delivery science, implementation science, patient centric, problem-driven research, socio-technical systems, systems engineering

## Abstract

**Introduction:**

Stated aims for digital healthcare transformation frequently cite goals for better coordinated patient-centric systems. However, despite advances in medical science, digital technologies, health policies, and billions of dollars invested over the past 25 years, most healthcare providers are far from fully realizing the demonstrated benefits of today's digital technologies for improving patient care. Sharing information across healthcare systems remains challenging. Problems with fragmentation, quality, inequities, and rising costs of care delivery persist. A recent study of 1,026 U.S. hospital systems found that only 15.8 percent achieved a digital maturity level needed to provide digitally enabled healthcare services to better coordinate patient care. More importantly, hospital systems that were most successful in digitally transforming demonstrated significantly superior patient outcomes. From a system engineering framework, we pose the problem as: How do we transform a system as complex as U.S. healthcare delivery from today's costly, fragmented provider-centric system to a better coordinated patient-centric system with improved quality, access, affordability, and patient and provider experience.

**Methods:**

This mixed methods, cross disciplinary research employs an integrative approach, synthesizing diverse sources of evidence to explore challenges and issues associated with healthcare digital transformation. Data extraction was performed by studying the full text of over 100 articles, case studies, and other sources, which were then analyzed thematically employing a “framework synthesis” methodology, which uses a deductive approach rather than the more common inductive synthesis approaches.

**Results:**

Research points to multiple factors impeding progress, not the least of which is the sheer complexity of the problem. Healthcare systems that achieved significantly superior results reported different approaches to digital transformation in ways that may not necessarily be apparent on the surface, primarily because it is not just about what they did but, more importantly, about how they did it. A growing body of knowledge indicates that achieving digital transformation requires substantially different systemic approaches to the problem that intersect across clinical, technical, behavioral, and organizational domains. In other words, systemic problems cannot be solved with siloed solutions.

**Discussion:**

This research explores these differences with the aim of determining how approaches differ, why the differences matter, and implications for achieving better results. Conceptualizing the healthcare delivery system as distinct from the practice of healthcare (medical practice) makes an important contribution to the evolving science of healthcare delivery – working *on* the healthcare delivery system versus working *in* the system.

## Introduction

Despite the current focus on the potential of artificial intelligence and digital transformation, recent research shows that few healthcare providers have been successful in fully realizing the potential benefits of today's digital technologies (1–5). Sharing information across healthcare systems remains challenging. Problems of healthcare fragmentation, quality, inequities, and rising costs of care delivery persist. Goals for a better coordinated, patient centric healthcare delivery system largely remain an aspiration despite advances in medicine, digital technologies, health policies, and billions of dollars invested over the past 25 years *as* recommended in the Institute of Medicine's seminal reports To *Err is Human* and *Crossing the Quality Chasm.* This is not a new problem. Rereading these seminal reports is a stark reminder of the lack of progress in addressing the inadequacies of the U.S. healthcare delivery system. Efforts at solutions to date appear to be as fragmented as the healthcare delivery system itself (3, 6–13).

Among a research study sample of 1,026 U.S. hospital systems (1), only 15.8 percent of hospitals had achieved a digital maturity level needed to provide digitally enabled healthcare services. Moreover, further analysis indicated that the digitally mature healthcare systems demonstrated significantly superior healthcare outcomes. Our study addresses the issue of why so few healthcare systems have fully embraced the potential of digitally enabled healthcare services despite the many demonstrated benefits. Research points to multiple factors, not the least of which is the sheer complexity of the problem (14, 15). Our research found that hospital systems which successfully achieved significantly superior results report substantially different approaches to digital transformation in multiple ways. This study explores these differences with the **aim** of determining how approaches differ, why the differences matter, and implications for achieving better results.

The “healthcare delivery system” encompasses a wide range of care givers and venues, all of which operate largely in silos leading to errors, gaps, duplication of services, and all too often, poor patient outcomes. The siloed, uncoordinated nature of healthcare across multiple specialties and settings, presents one of the greatest challenges limiting system optimization. The fundamental issue is how do we transform a system as complex as U.S. healthcare delivery from today's costly, inconsistent provider-centric system to a better coordinated patient-centric care system with improved quality, access, affordability, and patient and provider experience.

We propose a transdisciplinary systems engineering approach to the problem that intersects across clinical, technical, behavioral, and organizational domains. Thus, from an engineering perspective, we conceptualize a transformed healthcare delivery system as an *interorganizational system* that puts the patient at the center and supports collaboration and coordination across the continuum of care to improve outcomes, access, affordability, and patient and provider experience.

## A systemic problem

Today's healthcare ecosystem is clinging to two worlds: digital and paper, essentially layering expensive new technologies on top of outdated inefficient processes, contributing to increasing work and costs rather than reducing them. This failure to transition to updated digital capabilities is a leading contributor to burnout, increased workload, and care gaps (16). The failures generally are not in implementing the technologies but rather in implementing the necessary process, procedures, and workflow changes necessary to improve care delivery. Benefits are tied *not* to implementing the technology but to *applying* that technology to streamline *how* things are done: transforming workflows and work processes to better coordinate patient care. Essentially, this is not about changing the medical practice but rather about changing the system for delivering that medical care. Essentially, the explosion of scientific knowledge over the past 30 years has changed how medical professionals care for patients, but the structures and processes of most healthcare organizations are legacies of the past (4). Conceptualizing the healthcare delivery system as distinct from the practice of healthcare (i.e., medical practice) is significant and useful in rethinking how the delivery care system interfaces with patients and providers. Another way of thinking about this framing of the problem is working ON the delivery system as opposed to working IN the system. Consistent with the findings and recommendations of the 2001 Institute of Medicine (IOM) seminal reports *To Err is Human* and *Crossing the Chasm*, working on the healthcare delivery system is a systemic problem, not a problem of individual performance (17, 18). Systems theorists argue that traditional mechanistic models of organizations are insufficient for understanding complex healthcare tasks, advocating instead for viewing healthcare as Complex Adaptive Systems where outcomes emerge from cooperation across interconnected agents (19). Unfortunately, in the case of today's complex healthcare delivery system, dozens of providers all doing their jobs superbly well do not necessarily add up to superb results for patients, and sometimes even leaves patients still wanting for a diagnosis or solution for their health problem. Just as medical technologies have afforded a multitude of improvements in medical practice, digital technologies offer a multitude of new options for transforming the healthcare delivery system to better meet the needs of providers and patients.

The issue is important because evidence indicates a growing performance gap between healthcare systems with advanced digital capabilities in comparison to those lagging in making the transition. Findings of the recent Snowdon study of 1,026 U.S. healthcare systems indicated that those with digitally advanced capabilities had statistically significant reductions in infection rates and adverse events and significant improvements in surgical safety outcomes and overall quality and safety outcomes (1). Advanced digital capabilities are associated with more digitally enabled work environments with automated flow of data across information systems to enable clinicians and leaders to better coordinate patient care and to track quality and safety outcomes (1). Equivalent results have been demonstrated in other studies of digital capabilities as well (2, 20–23).

Several impediments present challenges in efforts to transform a system as complex as U.S. healthcare delivery from today's costly, fragmented provider-centric system to a better coordinated patient-centric care system with improved quality, access, affordability, and patient and provider experience. Not only are we dealing with a systemic problem where processes and workflows often intersect in multiple ways, of which not all care givers may be aware, but many of the necessary changes represent a major departure from long-established protocols. Some changes require a significant change in mindset (i.e., paradigm shift) for which the purpose or benefits may not be immediately apparent to everyone. These issues are further explored in subsequent sections.

## Methods

This mixed methods research employs an integrative approach, synthesizing diverse sources of evidence to explore challenges and issues associated with digital transformation in healthcare. The integrative approach is appropriate for this study as it allows for the inclusion of multidisciplinary research and other sources contributing to a more comprehensive understanding of the complex, multi-faceted nature of digital healthcare transformation (15, 24, 25).

The systematic literature review included sources spanning implementation science, user-experience (user-centered) design, design science, organizational development/transformational change science, socio-technical systems, medical science, translational science, implementation science, health informatics, healthcare delivery science (98), service science, and systems (or industrial) engineering. Sources in addition to peer-reviewed articles included use cases, industry publications, white papers, books, government reports (Example: ASTP/ONC publications), medical associations (Institute of Medicine, AHA, AMA, AMIA, Leapfrog Group, etc). A systematic search strategy was developed to identify relevant literature published between 2000 and 2025.

Inclusion and exclusion factors were clearly defined to ensure that the review focused on a broad cross-section of evidence that was most pertinent to the study objectives. The search itself was conducted over an extended period of time. Key search terms included “digital healthcare transformation,” “integrated care systems,” “digital health,” “patient-centric,” “patient-centered,” “user-experience design,” “design science,” “coordination of care,” “healthcare fragmentation,” “organizational or transformational change,” “digital transformation and healthcare,” “learning healthcare system,” “healthcare ecosystem.” Studies that focused on implementation or translation of solutions rather than solution design, patient-centric care, provided empirical evidence on outcomes, challenges, sustainability, and lessons learned, were prioritized. Relevant peer-reviewed articles and publications meeting inclusion criteria that were previously identified for a couple of the author's prior research projects were also included. Exclusion criteria involved studies that were not related to healthcare digital transformation, those that focused on a single technology without considering the broader integration, and those that did not address issues related to patient care and provider outcomes. Reference lists from selected research studies were cross-checked to identify relevant literature that may have been overlooked.

Data extraction was performed by studying the full text of selected articles and other sources, which were then analyzed thematically employing primarily the “framework synthesis” methodology, which uses a deductive approach rather than the more common inductive synthesis approaches. Framework synthesis uses an apriori “framework” informed by background material and team discussion to generate initial themes for analysis and refinement (26). Initial themes were studied and refined, taking into account varying perspectives from the lens of different disciplines or theories. Analysis also focused on not just what was done, but how it was done to the extent that details were provided. Insights into how things were done and what worked and why were often provided in discussion sections or “lessons learned” in use cases and case studies. For example, change management approaches were filtered through the lens of the most recent science on transformational change with special attention to strategies employed to foster new thinking about long standing problems, breaking out of old paradigms and cultivating changes in mindset; i.e. in practice and in approach to a problem.

This research also builds on the author's prior research into success versus failure and critical success factors in healthcare technology implementation, the changing role of executive leadership in clinical transformation, health information exchange, and healthcare delivery system implementation (12, 14, 27–36, 100). Assessing the significance of issues and relevant nuances was also tempered by the researcher's experience on multiple collaborative research projects working with clinical partners on the frontlines of care. Data extraction was performed by studying the full text of selected articles, use cases, and other sources, which were then analyzed thematically from a cross-sectional perspective.

### Impediments to progress: gaps in prior research and practice

Exploration of reported findings from healthcare transformation research and use cases surfaced several apparent gaps in prior research and in translation to practice. The significance of these gaps is discussed in this section and the implications for designing and implementing projects are discussed in the subsequent section on Results.

One significant impediment central to progress is a lack of clarity around what patient centric truly means and how that translates into practice for healthcare delivery. The term “patient centric” is widely used in the literature and in practice with a broad range of definitions, descriptions, and associated practices. Today's healthcare system coordinates care around providers NOT patients (2, 17, 37, 38). Digital transformation proponents would argue that until we can make that transition, we will not truly have patient-centric (or patient-centered) care (2, 37–40, 97). Thus, the issue becomes what impedes progress in moving in that direction, which has been advocated for over 35 years now, and how can digital information technologies support that transition.

### Patient-Centric care

The concept of patient-centric care is generally associated with the issue of quality of care. The Picker Institute pioneered a revolutionary methodological approach beginning in 1988 by directly asking patients, families, and recently discharged patients what mattered most during their healthcare experiences. This bottom-up methodology contrasted sharply with top-down provider or administrator definitions of quality care.

Through extensive empirical research documented in *Through the Patients' Eyes* (39), the Picker Institute identified eight dimensions that patients themselves considered essential to their care experience: 1) Respect for patients' values, preferences, and expressed needs. 2) ***Coordination and integration of care*.** 3) Information, communication, and education. 4) Physical comfort. 5) Emotional support and alleviation of fear and anxiety. 6) Involvement of family and friends. 7) ***Continuity and transition***. 8) ***Access to care***. This patient-derived framework represented a significant departure from healthcare quality metrics traditionally defined by clinical outcomes, provider performance, or institutional efficiency measures.

The Institute of Medicine incorporated this patient-centric framework into its influential 2001 report *Crossing the Quality Chasm: A New Health System for the 21st Century*. The IOM established patient-centeredness as one of six core aims for healthcare improvement, alongside safety, effectiveness, timeliness, efficiency, and equity. The report defined patient-centric care as “providing care that is respectful of and responsive to individual patient preferences, needs, and values and ensuring that patient values guide all clinical decisions.” (18) The IOM built upon the Picker Institute's empirical work, citing Gerteis et al. (1993) and noting that “The Picker Institute in Boston, Massachusetts, has been tracking patients' experiences in hospitals, clinics, and other settings since 1988” (18). In discussing dimensions of patient-centric care, the report referenced the Picker framework, though presenting a condensed version in some sections, while the full eight-dimension framework appeared in the source Gerteis work.

Critically, the IOM recognized that achieving patient-centric care required technological infrastructure: “The automation of clinical, financial, and administrative information and the electronic sharing of such information among clinicians, patients, and appropriate others within a secure environment are critical if the 21st-century health care system envisioned by the committee is to be realized” (18).

Healthcare thought leader Dr. Donald M. Berwick, former administrator of the Centers for Medicare and Medicaid (CMS), President Emeritus of the Institute for Healthcare Improvement, and Harvard Professor, has articulated patient-centeredness as fundamentally more radical than most healthcare rhetoric suggests. Rather than viewing it as improved customer service or bedside manner, Berwick frames patient-centric care as “a radical transfer of power from clinicians and the system to the patient” (38). In his view, genuine patient-centeredness means:

Control resides with patients: The proper source of control is the patient. A patient-centric system would put the patient in control of their care.
The system adapts to patients, not vice versa: In a patient-centric world, the patient's needs come first; the system bends to meet them, not the other way around.
Complete information transparency: All information: medical records, test results, clinical notes- belongs to the patient and should be fully and instantly accessible to them.
Customization as organizing principle: Care must be tailored to the patient's needs, values, and preferences. Anything less is not truly patient-centric.

While definitions of patient-centeredness as a theoretical ideal may vary, a fundamental challenge remains: Healthcare providers often operate within a systematically distorted understanding of patient experience. Research on patient shadowing where healthcare staff accompany patients through their care journey reveals a striking disconnect between provider and patient perspectives (41). For example, clinical staff perceive hospital wards as “crazy busy” environments, yet patients in those same settings experience prolonged periods where “nothing happens,” leading to profound loneliness, isolation, and anxiety (40). Another example is the revelation cancer physicians at Virginia Mason Medical Center experienced when they analyzed care pathways for ambulatory cancer patients. Using blue yarn to map the meandering journey throughout the cancer center—“to the lab on six, radiology on five, up to fourteen for the clinic, down to the infusion center on twelve, than across twelve to radiation” —the outcome “looked like some kind of abstract art as it went up and down one building then into another and up and down and around and then into yet another building then doubled back and wrapped around itself in horizontal and vertical lines.” (2) The experience raised questions about whether it made sense to force the sickest ambulatory patients to walk all over the large medical center for treatment or what opportunities this new insight might provide for improving the care experience for both patients and providers. This questioning led to a mindset change (paradigm shift) among Virginia Mason's redesign team resulting in major changes to facility construction plans.

The problem extends beyond simple misperception: healthcare professionals bring their own experiential “lens” shaped by clinical training, personal experiences, and professional detachment that systematically colors their interpretation of patient needs (40). As one participant reflected, staff wanted to provide companionship because “I hate to see or think of people being on their own and having no-one,” yet the very structure of care left patients feeling abandoned despite surrounding activity (40). This suggests that achieving truly patient-centric care requires more than policy mandates; it demands deliberate mechanisms to disrupt entrenched provider perspectives and reveal care as patients actually experience it. This is a transformational change of mindset (or paradigm shift). As one manager summarized the effect, shadowing gave staff “a thirst for quality improvements to improve patient experience,” and “as soon as you've been there, it makes it personal.” (40) The same issue arises when change is based on perceptions of academic researchers or IT analysts instead of the realities of care at the frontlines.

More recently, the concept of Integrated Care Systems (ICS) has gained attention as a strategic approach for making care more patient-centric (24). ICS is defined as “the coordination of services across the continuum of care, multiple service providers, settings, and levels of care to achieve improved patient outcomes, enhanced patient experience, and optimized resource utilization” (42, 43, 73, 74, 98). A recent study by Ebo et al. (24) identifies several core principles including patient-centeredness, where care is centered around the individual's needs and preferences; population health focus, addressing the health needs of entire communities; collaborative governance involving multiple stakeholders in decision making; and valued-based care that emphasizes outcomes rather than volume of services. According to Ebo et al. (24). ICS typically encompass primary care, secondary care, mental health services, social care, and public health functions, creating a unified framework for service delivery.

Beyond provider use of health IT, recent research shows that patients are now becoming significant users of health IT (44). This phenomenon is fundamentally changing patient expectations and the relationship between patients and providers. The outcome of this transition has been significant. Several studies on use of patient portals have shown promise in facilitating self-management across a number of critical health conditions by allowing patients' input and use of self-monitoring devices, facilitating communication, and delivering educational materials (44–49).

### Active, committed executive leadership

A frequently cited critical success factor for transformation initiatives is committed executive leadership (2, 21, 22, 24, 26, 28, 50–52, 100). However, a closer examination shows significant variance in how that leadership role is fulfilled. Evidence shows that the level of commitment, intensity, and hands-on involvement of CEOs and Boards of Directors in the most successful initiatives has been extraordinary. One such example is Virginia Mason Medical Center's transformation initiative chronicled in a book by Charles Kenney (2). Their approach was spearheaded by Dr. Gary S. Kaplan, Virginia Mason CEO with strong support of their Board of Directors. It is an extraordinary example of leadership, vision, and dedication in transforming from a deeply provider-centric to a patient-centric healthcare system, a more than 10-year journey. Central to the approach was the focus on the complex, systemic nature of today's healthcare delivery, and the necessity of working at the frontlines of patient care from both provider and patient perspectives. Recognizing the absence of any management framework for addressing systemic transformation in healthcare, leaders looked outside the healthcare system to the extraordinary quality results achieved by the Toyota Production System process (TPS), first studying it in depth and then adapting it for application in healthcare. Among the many concepts and methods transferable to healthcare were elimination of waste and inefficiencies, quality focus with zero error tolerance, focus at the frontlines of operation, and standardization of processes. Among lessons learned was the hard, hard work, perseverance, and long-term perspective required. Other critical challenges involved provider buy-in at all levels and understanding healthcare delivery from the perspective of the patient experience, which differed drastically from provider perceptions. The efforts and strategies for changing mental mind sets and helping providers envision possibilities for transforming the care delivery system, understanding the care delivery process as patients experience it, and implementing standards and procedures for eliminating errors and making the right thing the easy thing to do were extraordinary and cannot be understated. Moreover, the active role of the CEO with support of the Board of Directors were critical especially in addressing strong pushbacks from providers. Addressing problems actively at the frontlines was central to designing and implementing solutions.

Similarly, Kaiser Permanente's $4billion, 10-year journey in healthcare transformation chronicled by Dr. Louise Liang demonstrates the extraordinary leadership of CEO George Halvorson (21). Under the leadership of Dr. Halvorson and Dr. Liang, Kaiser Permanente's nationwide *HealthConnect* network has transformed care delivery with significant impact on patient care outcomes, efficiency, safety, and patient engagement and satisfaction. Research on success vs. failure (27–30, 54, 57, 100) also found that committed CEO leadership was one of the most dominant characteristics in effective implementation of the EHR Meaningful Use program.

### Significance of a problem-driven approach at the frontlines of care

The predominant approach to implementing changes in clinical practice has been translational science—designing a potential solution, testing it in a clinical trial environment, and then translating it to the frontlines of care. However, the reported success of this approach for uptake and sustainability of changes has proven to be low (8, 55). In contrast, healthcare systems reporting significantly superior results generally report taking problem-driven approaches bringing solution development closer to the frontlines of care and engaging users earlier in the process in both defining improvement targets and in shaping the solutions (21–23). Critical success factors reported in successful projects include clearly defined problems, executive leadership, user buy-in and involvement, physician leadership, and well-defined patient outcomes (Not just process or institutional outcomes) (14). It is all too easy to assume everyone understands the problem since they are living with it every day. However, given the complexity of our healthcare delivery system, shortcutting the process of getting to the heart of the problem and understanding how it impacts patient care at the frontlines, can be highly consequential because in many ways, how the problem is framed, predetermines the set of potential solutions. Moreover, when solutions are developed remotely by technical experts, managers, or providers, they are often insensitive to patient perspectives and how patients experience the care pathway. A distinguishing point among healthcare success stories is the amount of time and effort invested at the outset in engaging cross-cutting stakeholders at the frontlines in studying the problems, developing explicit targets for improvement, and bringing a new mindset to developing solutions. It turns out that designing a solution for zero error tolerance, for example, does not elicit the same thinking as designing for 98 percent tolerance. In other words, a solution designed to allow a 2 percent error tolerance is very likely to produce precisely that result (2, 21–23, 56–58). Research by Andrews, Pritchett, and Woodcock at Harvard Kennedy School (56) provides insight into how a problem focus in project design and implementation can ensure that the focus is solving specified problems as the goal (rather than introducing a pre-designed solution) as well as allowing continuous changes in the design to ensure the problem is effectively addressed. Moreover, according to Andrews, a problem-driven approach promotes a search for alternatives that offer a solution (rather than just providing new ways of doing things). They suggest that many projects claiming to be problem-driven, are not problem-driven at all, and thus “their strategy has no real means to draw attention to the need for change, provide a rallying point for coalition building, or offer a ‘true north’ destination of ‘problem solved’ to guide, motivate and inspire action” (56).

### Health information technology, including AI, as an enabler or catalyst for change

It is widely recognized that information technology is not the solution, but rather an enabler, or a catalyst, for designing new approaches for solving long-standing problems. Nonetheless, when planning digital transformation projects, healthcare institutions all too often allocate 90% of the resources to implementing the technology with little time, effort, and resources devoted to establishing clinical objectives or transforming care delivery practices. The data shows that less than 0.1 percent of the total $3 billion plus spend on healthcare annually is currently devoted to research designed to improve how we deliver healthcare (58, 59). Multiple factors contribute to this failure including the previous discussion about lack of clarity around framing the problem and designing solutions. Moreover, technology implementation is far more tangible and less complex than transforming care delivery processes (5, 24, 60). It is far too easy to assume that making technology available will automatically lead to innovation, which in fact is seldom the case—especially in healthcare where solution development almost always intersects across multiple fragmented care pathways and silos of operation. Our research shows that healthcare institutions achieving transformation goals invest at least as much, if not more, in designing and implementing transformation initiatives as they do on technology implementation (2, 14, 21–23, 27, 28, 61, 62).

### The power of healthcare information sharing for care coordination and integration

The need for health information technology interoperability and data sharing is an essential component of the 2001 Institute of Medicine's *Crossing the Quality Chasm* recommendations: “The automation of clinical, financial, and administrative information and the electronic sharing of such information among clinicians, patients, and appropriate others within a secure environment are critical if the 21st-century health care system envisioned by the committee is to be realized.” (18) Yet here we are more than 25 years later still struggling to make these digital capabilities a reality. After multiple earlier attempts, a national TEFCA (Trusted Exchange Framework and Common Agreement) health information network finally became a reality in December 2024. (53, 63) TEFCA is designed as a network of networks to provide the technical standards, capabilities, and governance structures needed for digital health information exchange nationwide. Concurrently, newly developed U.S. Core Data for Interoperability (USCDI) standards were also released. FHIR standards for application interfaces continue to evolve as well and are now supported by TEFCA. Development and expansion of all three continue, but resistance to adoption remains high. Why the delay and why the importance?

Globally, organizations such as the World Health Organization have developed frameworks like the SMART guidelines (Standards-based, Machine-readable, Adaptive, Requirements-based, and Testable) to support the uptake of digital health standard and interoperability, emphasizing stakeholder engagement and customization to local contexts as critical success factors (64). The World Health Organization also has developed Digital Adaptation Kits (DAKs) as a software-neutral mechanism for translating clinical guidelines into standardized digital formats that support interoperability and quality assurance in the transition from paper to digital systems (65).

Despite a continuing focus and incentives to promote widespread information sharing, our research suggests general low awareness of the potential benefits and the power (affordances) of information sharing as an essential foundation for making care more patient centric by improving care coordination and integration (66–68). Although information sharing as the foundation for coordination is long-established in other industries—banking, airlines, shipping, and manufacturing supply chains, for example—in healthcare delivery it runs counter to current paradigms (or mindsets) about safeguarding patient data and privacy. The potential for redesigning workflows and improving care pathways, intersects across multiple operational units and often multiple medical practices or institutions. Efforts may also be undermined by the sometimes-perverse financial incentives of the U.S. third-party payer system. It is not a coincidence that many of the healthcare systems that have been most successful with their transformation efforts have been integrated care systems, and thus they have more directly benefitted financially from improved outcomes and efficiencies (42).

Several leading healthcare institutions have demonstrated the power of information sharing for care coordination and integration and achieving more patient-centric care pathways. Nonetheless, implementation remains more of an exception than a reality for most healthcare institutions (2, 21–23, 26, 52). A 2019 study by Poku, Kagan and Yehia found that only 7% of healthcare executives, clinical leaders, and clinicians indicated that their patients' care is fully coordinated across various health settings (66). While the need for integrated care, portability of health information, and alignment of value incentives across stakeholders have been widely recognized, only a handful of organizations have been able to implement aspects of these goals within their health systems and fewer still have been able to integrate with outside organizations (66).

### User-centered design science at the frontlines of care

Addressing digital healthcare transformation from a systems perspective raises several considerations. The IOM reports (18) clearly differentiate problems with the healthcare delivery system as a “systems problem” rather than a problem of individual performance, but what are the implications for designing and implementing solutions? Addressing this question intersects with other healthcare considerations as well. A prime consideration is healthcare standards of evidence-based medicine. Implementing IT-based changes in workflows or protocols at the frontlines of care, generally requires appropriate research methodologies to validate the impact on patient outcomes. Changing who does what during the care pathway might also intersect with professional licensing practice parameters or other policy or regulatory requirements. For example, telehealth has run into multiple licensing issues related to practicing across state borders as well as payer issues.

Tools and frameworks for user-centered design, including human personas and human behavioral modelling, have been well-established in engineering disciplines (69) and offer potential methodologies for healthcare to adapt in designing more patient-centric delivery systems. However, research also shows that user-centered design principles, while widely espoused, are often not effectively employed in digital health initiatives, with common shortcomings occurring at all stages of the design process (70). Our research suggests that institutions that took a more team-based, iterative design science approach to transformation were far more likely to achieve intended performance outcome goals (1, 2, 13, 21–23, 38, 57, 61, 71–75). Design science differs from more traditional translational science approaches in multiple ways that appear to be consequential in achieving results. For example, it brings the transformation initiative to the frontlines of care from the outset of the design process rather than at the later stages of the implementation process. Building buy-in and engaging providers at the frontlines early in the process was often cited among “lessons learned” as important to reported successes. In-depth use case evidence offers insightful examples of how the dynamics of the process can lead to new thinking and provider buy-in.

Design science aligns with the IOM concept of the Learning Healthcare System (LHS) (58, 76, 77). LHS can be seen as an evolution of the earlier notion of “knowledge translation and exchange” that fuses research and healthcare delivery by embedding advanced research methods within the context of everyday care (78, 79). The concept of the learning healthcare system was first conceptualized and promoted by the Institute of Medicine in the early 1990's (18, 80). The original concept was subsequently expanded by conceptualizing that LHSs operate at three nested levels: (a) the micro-clinical level, where learning activities center on using best evidence for clinical decision-making by providers and patients; (b) the meso-organizational level that focuses on generating and using evidence on health service delivery arrangements and population health; and (c) the macro-system level where the focus is on policy making including resource allocation and regulatory requirements (81).

“A comprehensive LHS approach is particularly relevant in the complex multicomponent changes to how healthcare is organized, financed, and delivered” (79). While the concept of LHS is broadly supported in the healthcare community, guidance on the best practices for operationalizing the concept in practice remains underdeveloped (79). “Existing LHS models and frameworks lack practical detail about how different streams of evidence and research methods intersect in the learning activities, and where (and with whom) relationships between researchers and health system operators are formed and sustained” (79). More broadly, these findings suggest that there is need to revisit existing project management frameworks and methodologies to better address the complex dynamics of implementing digital transformation. This issue is explored further in a later section of the paper.

### Distinctive challenges of implementing transformational change

Understanding about the organizational change management process itself has been changing, especially in the domain of digital transformation. Researchers have identified several distinctive challenges beyond the dynamics of the generally accepted practices of change management theory (57, 75, 82). Most of the relevant research about digital transformation comes out of the social sciences research on organizational psychology/organizational behavior (OB) and organizational development (OD) rather than the current body of technology literature on technology acceptance. Fundamentally transformational change occurs not just at the organizational and operational levels, but even more significantly at the cultural level, changing the underlying dynamics of organizational culture.

Working at the level of transformational change, involves three critical and unique dynamics: (1) the future state cannot be fully known in advance; (2) significant change in organizational culture and in individual mindsets and behaviors are required, and (3) the change process itself cannot be tightly controlled and is difficult to manage because outcomes are uncertain, and the human dynamics are too unpredictable (75). These unique dynamics present many challenges for working within traditional scientific research methodologies and institutional management infrastructures. Addressing these challenges requires a new mindset, with a whole-system, long-term, process perspective. Transformation is not something that can be led apart from where real “work” happens (i.e., the frontlines)—it must be intertwined, embedded, and integrated into activities and infrastructure that drive action in healthcare organizations (75). Transformational change is a long-term process—7–10 years. The three unique dynamics identified by Anderson and Anderson are clearly evident in the Virginia Mason transformation initiative with multiple examples of strategies for addressing them (although there is no direct reference or correspondence between the two books).

Research around Affordance Theory is also relevant, albeit, not as directly. First introduced by J.J. Gibson, affordance theory suggests that the possibilities for action inherent in an object or environment are directly perceived by an individual, depending on both the object's physical properties and the user's own capabilities or objectives. The concept suggests that the design of an object should align with a user's needs and abilities to create intuitive and accessible experiences. While initially focused on physical environments, the concept was extended for digital environments by Donald Norman to application in Human-Computer Interaction (HCI) for user interface design. In these contexts, perceived affordances become critical, as designers must ensure that users can correctly interpret the intended actions, especially in the absence of physical cues. In healthcare, affordance principles can be useful in providing insight and underscoring the importance of creating products and environments that are clear, intuitive, and easy to use. Conceptually, in the case of transformational change, it is the affordances for changing the system paradigm that are of prime interest.

## Results: implications for healthcare transformation—how dynamics make a difference

The previous section identifies several ways in which the approaches to digital transformation among healthcare systems achieving digital maturity with significantly superior results differ from most of today's healthcare systems. This section expands the discussion about why these differences are consequential and the implications for developing more productive avenues for moving forward. Healthcare transformation initiatives face many challenges. Methodologies are not well defined and lack frameworks or common language. Initiatives generally involve a broad range of stakeholder expertise, experience, perceptions and mindsets. Thus, getting everyone on the same page and singing off the same sheet of music is a challenge. In many ways, the journey is more akin to leading an orchestra trying to create harmony than following a structured project management methodology. It is also helpful to keep in mind that no one designed today's healthcare system. And many would argue it is not a system at all (4, 6, 25). Healthcare delivery has drifted into today's fragmented system over time as advancements in medical science and technology have created increasingly greater specialization, essentially moving from the family doctor making house calls to today's ecosystem of highly fragmented care. Moreover, it is a system optimized for acute care, at a time when there is a rapidly growing need for managing the health of our aging population with multiple chronic conditions, which now account for 75% or more of healthcare costs (83–85).

This section begins with a discussion about the significance of transitioning from a translational science to a design science approach to transformation and then continues with discussion of other relevant factors.

### Transitioning from a translational science to a design science approach

Transformational change requires well-planned orchestration focused simultaneously on problems that intersect across clinical, technical, behavioral, and organizational domains. Approaches must consider both the affordances provided by new technologies as well as the changes in mindset and organizational culture needed to gain buy-in and commitment of major stakeholders (i.e., changing the paradigm of the care delivery system). A preponderance of evidence from healthcare transformation initiatives suggests that a critical success factor has been moving the implementation process to the frontlines and engaging users early on in problem analysis, solution design, and implementation. For example, there are consequential differences in engaging stakeholders in advisory roles verses engaging them directly from the outset of problem analysis, making the stakeholders the advocates for change (56, 86). People support what they help to create. Importantly, developing a deep understanding of the problem becomes a motivating factor for the next stage of designing solutions (57, 75). Even more importantly, when problem analysis at the frontlines encompasses patients and how they experience care pathways, it can be eye opening for providers, helping to focus their improvement efforts (40). As previously pointed out, research using patient shadowing often reports how surprised providers were about the wide disparity in viewpoints.

Our research findings also indicated that iterative approaches to design and implementation frequently incorporated the IOM learning healthcare system (LHS) concept with feedback loops supporting a continuous improvement process (22, 77). A comprehensive LHS approach is particularly relevant in the complex multicomponent changes to how healthcare is organized, financed, and delivered (78, 79).

### Ecosystem perspective (i.e., whole system approach)

Research shows that institutions realizing the greatest benefit from new technology take a far more integrated approach from a holistic, ecosystem perspective (2, 28, 74, 87–89). An ecosystem approach takes into consideration people, process, technology, and structure. This perspective makes complex contexts such as healthcare more understandable by applying system-level thinking (9, 61, 90, 91). Systems theory frameworks, such as the Neuman Systems Model, provide theoretical approaches to addressing the “wicked problems” inherent in integrated care design—problems characterized by unclear, unpredictable, and incomplete data (92). Ecosystems are dynamic, constantly changing on multiple system levels (micro, meso, and macro). While these levels are different, they are interdependent within the whole system. Thus, changes in one unit or level have ripple effects (sometimes unanticipated) across other levels. The implication is that making changes in one unit or level requires corresponding change on other levels to achieve the anticipated benefits. This is a major gap in the prevalent model of siloed disciplinary innovation, which often results in failure to attain sustainability (14, 99). From a patient perspective, care pathways often cross multiple silos spanning multiple, sometimes competing, healthcare institutions.

### Problem-driven: starting with the end-in-mind

Digital transformation starts with cultivating new thinking about old problems. Thus, a problem-driven approach is recommended because it provokes reflection, mobilizes attention, and promotes targeted and context-sensitive engagement (56, 93). A problem driven approach also can be empowering for users. It is important to recognize, however, that leaders cannot change the thinking of others—a reality that can be difficult to acknowledge in organizational settings where leaders are accustomed to directing the goals and activities of their employees (57). Leaders can only facilitate changes in thinking by creating an environment and experiences that prompt individuals to challenge their thinking and consider new options (57). As the age-old adage goes: You can lead a horse to water, but you cannot make him drink.

Surfacing the underlying thinking about current processes or problems was a critical starting point discussed in almost all the in-depth cases we studied. The specific approaches varied, but the objectives were similar: Probing to gain insight into the root cause and seeking to identify system performance deficiencies that cannot be ignored and that matter to key stakeholders. One of the most powerful ways to identify opportunities employed by transformational leaders was to listen to the patient's perspective (2). Some healthcare systems, for example, assigned healthcare workers to silently shadow a patient through their entire care pathway for diagnosing and treating a healthcare problem (2). Talking with patients and listening closely for the challenges they face can be enlightening and develop a deeper understanding of the patient's experience. It is important to keep in mind also that new solutions don't always mean throwing out past practice. Sometimes it is a matter of modifying or expanding on current practice—an approach that Ayars & Clifford (57) refer to as “And Thinking.”.

Starting with the end in mind was commonly identified as a critical success factor among healthcare transformation leaders (94). Another critical factor that set most successful transformation initiatives apart was the clarity with which they articulated patient outcome goals along with their metrics for tracking progress (2, 21–23). In other words, what will, or should, the problem look like when it is solved, and what will be the benefits for patients and providers?

### Team-based healthcare delivery

The team-based healthcare delivery model has been gaining support as an important strategy for improving care coordination and transitioning to patient-centric care (13, 95). Once again, moving to team-based care represents a major paradigm shift from long-established norms. Team-based care is designed to enhance efficiency and access, improve quality of care, increase satisfaction for all involved in medical care, patients, providers, and staff.

 (95) Teams are generally led by one or more physicians and comprised of multiple clinical staff such as registered nurses, physician assistants, licensed practical nurses, medical assistants, population health navigator, and sometimes clinical pharmacists, or social workers.

Although often promoted as a strategy for improving care coordination, the literature and examples suggest that it is far from patient-centric. For example, guidelines for designing team-based care published by the American Academy of Family Physicians (AAFP) (95) are predominantly organized around optimizing performance of care providers. Thus, although the strategy offers significant potential for improving care delivery, it perpetuates the current paradigm of provider-centric care and it could be misleading to assume that it was advancing goals to make care more patient-centric. However, that does not mean necessarily that a care team approach could not be designed to be optimized around patient needs. The AAFP does advocate for the importance of practice transformation and workflow innovation and redesign and incorporation of technologies such as telehealth, which affirms that potential for evolving into more patient-centric models. Once again, however, it cannot be assumed that team-based models automatically lead to patient-centric care in the absence of intentional goals, strategies, and resources for achieving that aim. A key component for team-based models is a comprehensive digital information system to support care coordination, provide clinical decision support, and prevent mistakes. Ensuring that all team members always have access to the most current patient information and that handoffs from one team member to another are seamless is critical.

### Integrated care. Bringing the care to patients instead of patients to the provider

The concept of bringing care to the patient may seem radical at first blush. However, in essence this model is already in many ways the practice for acute hospital care where providers come to the bedside. With technological advances many specialty services can be brought to the patient bedside, such as with the use of portable x-ray equipment or other imaging technologies and EKG tests. However, an often-missing piece is a lack of communication across the different providers involved in the patient's care. Bringing care to the patient is far from common practice for out-patient care (i.e., ambulatory care), however, where patients may travel far and wide to different specialists and specialty care facilities. Nevertheless, if we think historically back to simpler times where a family doctor made house calls carrying his toolkit with him, we might view it as “back to the future.” Patient medical history was one patient-one record, with family history encompassing multiple generations. As medical science has advanced, healthcare has become increasingly complex and specialized, but also increasingly fragmented, error prone, and costly. Increased quality issues and errors are often attributed to the multiple handoffs across fragmented care specialties and services. With the growing population of aging patients living with multiple chronic conditions, it raises the question: Does it realistically make sense to require the sickest of patients to run all over the place for care? Especially given that many may be driving partially impaired or reliant on finding others to drive them. Can we do better?

**Figure 1:**
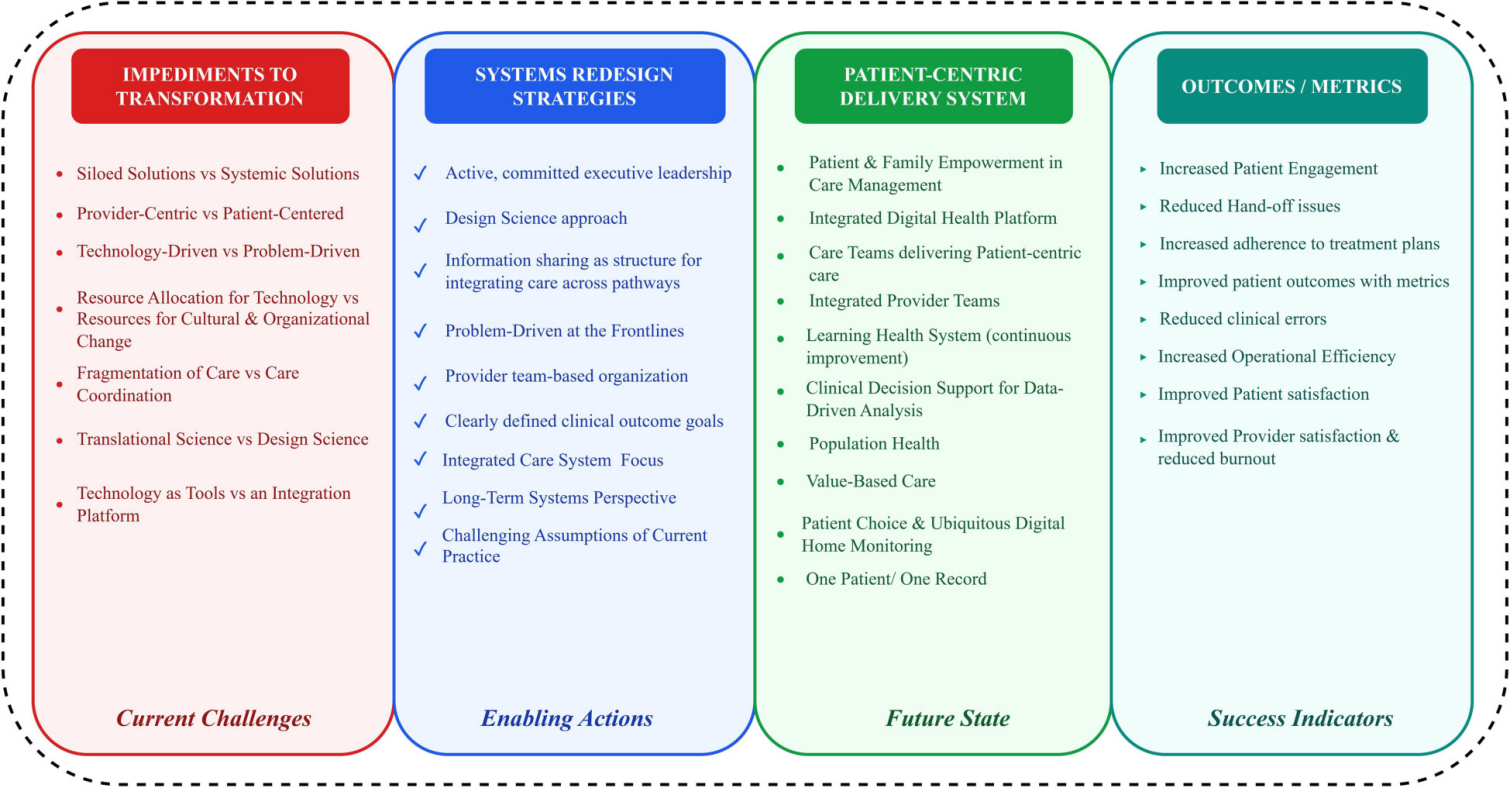
Patient centric integrated healthcare delivery system.

In Figure 1, we provide a conceptual framework for thinking about managing digital healthcare transformation from a systemic perspective with a focus on making it more patient centric. How are we defining patient-centric? What are the impediments to making the transformation from today's fragmented provider-centric ecosystem? How do our redesign strategies and research need to change? And what are our outcome goals?

## Recommendations for healthcare delivery system transformation

As readers may have noted in the preceding discussion, many of the gaps and issues discussed are rather nuanced. This is one of the characteristics of transformational change that make it difficult to differentiate success from failure: why similar projects succeed in one organization or context but fail in another. It is fundamentally not differences in *what* is done but rather *how* it is done. Thus, on the surface, especially at a high level, a project plan or action plan may appear very similar when, in fact, the way organizations carried the plan out is very different in ways that are not immediately apparent. These differences generally address the factors that address change at the level of system dynamics and organizational culture.

The term *digital healthcare transformation* is widely used nowadays in the context of quality improvement, but its meaning varies and the dynamics for achieving it are not well-understood. The underlying concept is that the ability to harvest the power (i.e., realize the affordances) of advanced information technologies requires much more than a collection of technology projects. It implies a systems level approach to improving patient outcomes from a healthcare ecosystem perspective that recognizes the interdependencies across the healthcare delivery system.

Our research points to multiple factors impeding progress in digital healthcare transformation, not the least of which is the sheer complexity of the problems and competing interests within our current third-party payer healthcare delivery system. “U.S. healthcare delivery system epitomizes a complex, evolving transdisciplinary domain intersecting information systems, policy, economics, and public health” (15, 96). It involves intricate interactions between stakeholders, including patients, healthcare providers, insurers, policymakers, and technology developers and encompasses the need to balance quality of care, accessibility, cost-effectiveness, and technological innovation (15). Thus, the notion of being able to transform everything on all levels is admittedly exceedingly ambitious, if not unrealistic. Nonetheless, a system approach to transformational change is still critical to success. Improvements implemented in silos have little staying power. A common characteristic among healthcare institutions that have been successful is an overall delivery system strategy with well-defined goals for improved patient outcomes. Redesign strategies for digital transformation need to be patient focused and encompass the entire care pathway as experienced by patients, aiming to integrate across the long-established organizational silos.

Achieving transformational change in healthcare requires a high level of digital maturity as well as substantially different approaches than typical technology implementation and change management methodologies. These differences are not well recognized or understood. Further implications of these differences, identified in prior sections, are discussed here.

**Figure 2:**
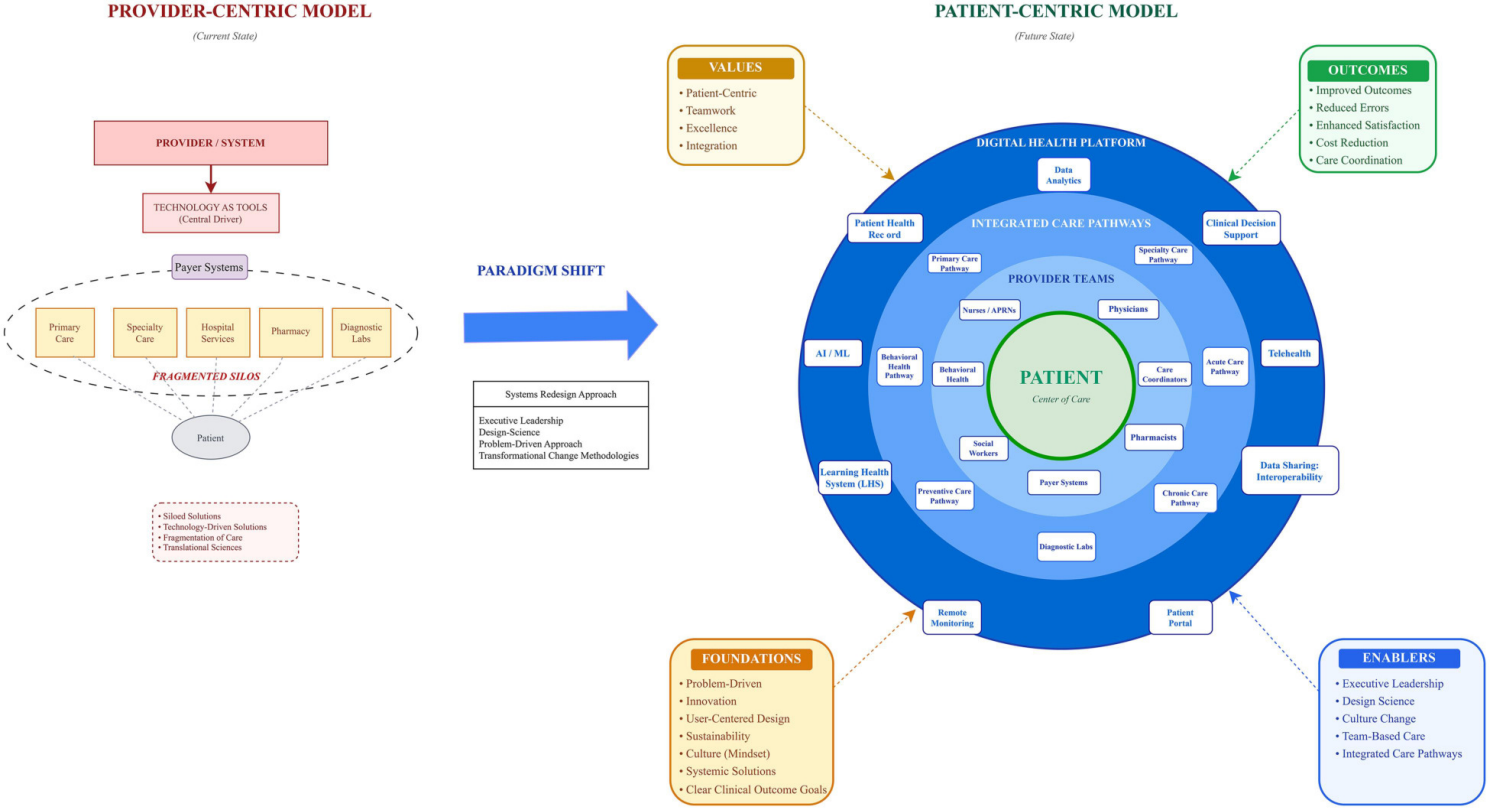
Healthcare delivery system transformation.

Our recommendation for framing the Healthcare Delivery System as a distinct concept (healthcare delivery science) is especially useful in clarifying the focus and intent of transformation strategies. Transformation requires taking on a medical culture that by nature is far more often provider-centric than patient-centric; a culture instinctively rooted in silos, resistant to teamwork, standardized work concepts, and quite often, evidence-based care (2). While clinicians readily buy into to the goals for improving quality, reducing variation, and preventing mistakes, they frequently push back with concerns that standardizing work somehow robs clinicians of freedom and creativity. Yet, results demonstrated by the most successful innovators consistently show that well-defined standards and procedures can improve quality, reduce safety hazards, and free up time for clinicians to take better care of patients—and use their creativity more fully (1, 2, 8, 21, 22, 52). For example, after a series of foundational delivery system changes, Virginia Mason increased the amount of time that nurses focus directly on patient work from approximately one-third to nearly 90 percent (2). The graphic in Figure 2 seeks to help clarify the distinction between standardizing medical practice from standardizing the information systems, workflows, interoperability standards, data standards and other structures that support healthcare operations and the work of care providers.

Thinking about the healthcare delivery system as a distinct concept also helps in developing a deeper understanding of differences for delivering care in different healthcare settings. This distinction is especially relevant in the case of Acute care vs. Chronic Care. Whereas the dominant care delivery model in most healthcare systems is designed around acute care, the need for chronic care has been exploding, driven by an aging population with multiple chronic conditions. This mismatch and the lack of chronic care models have become major issues for care delivery. Moreover, critical differences in healthcare delivery system requirements between acute care and chronic care are not widely understood, especially related to the importance of the need for more patient-centric care coordination across often complex care pathways spanning multiple institutions.

Based on the findings of our research, the graphic in Figure 2 frames the healthcare delivery system as an integrated patient-centric care system in contrast to today's fragmented provider-centric healthcare ecosystem. It envisions an interorganizational system integrated from a patient-centric perspective though the power of information sharing and an integrated healthcare technology platform. In the graphic, we visualize an advanced digitally transformed healthcare delivery system as an integrated platform for supporting medical practice and enabling a transition to better coordinated patient centric care. Digitally advanced systems can streamline workflows and make available the power of information sharing as a tool for coordinating patient care across multiple care pathways. It allows for integration of information and services enabling seamless integration across telehealth services, imaging services, laboratory services, pharmacy services, and more. It would provide a care platform that facilitates patient information sharing at the point-of-care, wherever that may be, ensuring that the most up-to-date relevant information is always available. A platform that facilitates bringing care to patients rather than having patients chasing care providers. A platform that supports population health management, risk assessment, and pay-for-value care models as well as timely claims processing. A digitally advanced system that improves the healthcare experience for both providers and patients. Underlying the healthcare delivery system are the foundational concepts or strategies critical for transforming and sustaining the delivery system and the Values that underpin them.

Transitioning from today's provider-centric delivery model to a patient-centric model represents a significant healthcare paradigm shift and raises many issues. From whose perspective will patient centric be defined? The Provider or the Patient? Research results, using methodologies such as patient shadowing, show a surprisingly wide divergence in perspectives in understanding the continuum of care from the perspective of the patient's journey vs. the provider experience.

Should outcome goals for transforming workflows and work processes to better coordinate patient care focus on patient outcomes, process outcomes, or institutional outcomes? Research supports the importance of focusing on patient outcome results (not activities or process measures). What were the patient's goals for the visit and how well were they met? Will patient pathways be redesigned with a people-focus or a disease-focus? For example, all diabetic patients vs. a diverse panel of patients assigned to a specific care team. To what extent will information systems be designed to capitalize on the power of information sharing as a foundation for care coordination (interoperability remains a major hurdle). How might a delivery system model be redesigned to bring care to patients rather than bringing patients to providers? Would one model fit all or are different models needed for different care needs? It is telling that current published models and guidelines for transforming to team-based care are organized with far more attention to provider experience than patient experience, even though the goals are better coordination of patient care (Hopkins).

The critical role of CEO Leadership and Board leadership for digital healthcare transformation cannot be over-emphasized (2, 23, 28, 38). Recognized healthcare leaders go so far as to say that if the CEO is not personally committed to the work and perseverance required to lead the effort, a healthcare organization should not undertake the challenge of healthcare delivery system transformation (38). Managing transformational change is a long-term, iterative process requiring a unique skill set which clashes with the prevailing short-term focus on results.

Conceptualizing the healthcare delivery system as distinct from medical practice and viewing it from a systems engineering perspective provides a helpful framework for addressing the exceedingly complex issue of healthcare delivery system transformation. Working ***on*** the system vs. working ***in*** the system. It provides a framework consistent with the IOM seminal reports' central framing of healthcare delivery failures as a system problem and not an individual performance problem. From a provider lens especially, it helps facilitate a change in mindset that can visualize “delivery system optimization” as distinct from “medical practice” although highly interdependent, thus changing the conversation.

Digital transformation of the U.S. healthcare system is a highly ambitious vision given the sheer complexity of the healthcare delivery system. Nevertheless, when we look back at the impact of the Internet since it first became available for commercial use in the 1990's, we can readily see the transformational power of information sharing on a global scale in industries such as banking, airlines, and manufacturing supply chains. Our research revealed many examples of significant improvements in patient outcomes achieved through improved information sharing and patient centric redesign of care pathways. The healthcare institutions that have developed the digital maturity levels and leveraged them to transform care delivery are leading the way. The effort is still a work in progress, but their stories provide critical insights into what differentiates success from failure in healthcare digital transformation initiatives.

## Future directions and limitations of the study

An increasing number of researchers across disciplines are beginning to question the wisdom of continuing to invest in siloed solutions to the systemic challenges of healthcare transformation (5, 54, 60). Researchers cite the low uptake rates, poor record of sustainability, and lack of evidence on the comparative value of emerging innovations (5). Much of the literature appears to conflate the concepts of innovation and transformation with the terms often seemingly used interchangeably. Innovating a new way of doing the same old thing is not necessarily transformative. Whether capturing patient information on paper or digitally does not transform anything unless associated changes in process and workflow also change. Moreover, it increases the work when the information is then printed out and given to the provider on paper. Another way of framing the problem is layering expensive new technology on top of old, inefficient paper processes.

Conceptualizing the healthcare delivery system as distinct from the practice of healthcare (medical practice) makes an important contribution to the evolving science of healthcare delivery (also called implementation science or translational science). It recognizes the systemic nature of transformational change and aligns with the IOM concept of the integrated learning healthcare system. The proposed systems engineering approach intersects across clinical, technical, behavioral and organizational domains. It also bridges the gap between invention or innovation in the lab and translation (dissemination)—or lack thereof—into practice at the frontlines of care by supporting design science approaches that focuses on the patient experience and improved patient outcomes (patient-centric). Recognizing that there are no one size fits all solutions to transformational change, the proposed approach provides a framework for each healthcare institution to tailor solutions to their unique needs while guided by proven fundamentals for success.

More research is needed on appropriate methodologies for healthcare delivery transformation on a system level. The emerging science of healthcare delivery is still in its infancy. Early leaders are forging the way, but the time, work, and resources required are extraordinary and all too often beyond the capacity of smaller healthcare systems. Although each healthcare system must forge their own pathway and experience their own learning curve, how might they benefit from the experience of those who have gone before? How might emerging patient-centric care models be shared? What organizations or institutions might provide guidance and how would it be funded? For example, in response to a growing number of requests, Virginia Mason established an educational institute in 2008 to share their experience with healthcare transformation. Might university researchers collaborate with healthcare institutions to explore new pathways? Might these projects be supported by funding agencies such as AHRQ, PCORI (97), or NIH to achieve both tangible results and publishable research? How might the prevailing project management and change management methodologies be modified or reinvented for leading transformational change? Recognizing the long-term magnitude of the transformation challenge, progress will come in stages, but little if any information is available, for example, about establishing priorities for where to begin or how to sequence phases. Much work remains to be done.

Given its complexity and transdisciplinary scope, this research cannot hope to be comprehensive. The literature and other sources studied represent a broad sampling of relevant research, but it is highly likely there is research not included that could provide additional insights. However, while noting these limitations, one thing can be said with confidence. The importance of digital healthcare transformation cannot be overstated, but so far, the research to guide this process is limited.

This research brings a system engineering perspective to the body of knowledge on digital healthcare transformation. It analyzes differences in approaches used by healthcare institutions that have achieved significantly improved patient outcomes in comparison to less successful attempts. The findings discuss how approaches differ, why the differences matter, and implications for achieving better results. Conceptualizing the *healthcare delivery system* as distinct from the practice of medicine is proposed as a useful extension of healthcare delivery science in helping to merge practice and research at the frontlines of care where ultimately the solutions must reside. Conceptualizing a transformed delivery system from the perspective of an *interorganizational system* that puts the patient at the center and supports integration across the continuum of care offers fresh perspectives for transforming today's fragmented care system to better coordinate patient care—a paradigm shift to a better integrated healthcare system to improve outcomes, access, affordability, and patient and provider experience.

## Data Availability

The original contributions presented in the study are included in the article. Further inquiries can be directed to the corresponding author.
